# Correction: SLiMEnrich: computational assessment of protein–protein interaction data as a source of domain-motif interactions

**DOI:** 10.7717/peerj.5858/correction-1

**Published:** 2018-11-21

**Authors:** Sobia Idrees, Åsa Pérez-Bercoff, Richard J. Edwards

**Affiliations:** School of Biotechnology and Biomolecular Sciences, University of New South Wales, Sydney, NSW, Australia

Corrigendum:

After publication, the authors noticed an error in the title that was introduced at the typesetting stage. The title has been amended to: “SLiMEnrich: computational assessment of protein-protein interaction data as a source of domain-motif interactions”. Similarly, “SLiM-Enrich” has been corrected to “SLiMEnrich” throughout the article.

